# Parallel-META: efficient metagenomic data analysis based on high-performance computation

**DOI:** 10.1186/1752-0509-6-S1-S16

**Published:** 2012-07-16

**Authors:** Xiaoquan Su, Jian Xu, Kang Ning

**Affiliations:** 1 Qingdao Institute of Bioenergy and Bioprocess Technology, Chinese Academy of Sciences, Qingdao, Shandong, China

## Abstract

**Background:**

Metagenomics method directly sequences and analyses genome information from microbial communities. There are usually more than hundreds of genomes from different microbial species in the same community, and the main computational tasks for metagenomic data analyses include taxonomical and functional component examination of all genomes in the microbial community. Metagenomic data analysis is both data- and computation- intensive, which requires extensive computational power. Most of the current metagenomic data analysis softwares were designed to be used on a single computer or single computer clusters, which could not match with the fast increasing number of large metagenomic projects' computational requirements. Therefore, advanced computational methods and pipelines have to be developed to cope with such need for efficient analyses.

**Result:**

In this paper, we proposed Parallel-META, a GPU- and multi-core-CPU-based open-source pipeline for metagenomic data analysis, which enabled the efficient and parallel analysis of multiple metagenomic datasets and the visualization of the results for multiple samples. In Parallel-META, the similarity-based database search was parallelized based on GPU computing and multi-core CPU computing optimization. Experiments have shown that Parallel-META has at least 15 times speed-up compared to traditional metagenomic data analysis method, with the same accuracy of the results http://www.computationalbioenergy.org/parallel-meta.html.

**Conclusion:**

The parallel processing of current metagenomic data would be very promising: with current speed up of 15 times and above, binning would not be a very time-consuming process any more. Therefore, some deeper analysis of the metagenomic data, such as the comparison of different samples, would be feasible in the pipeline, and some of these functionalities have been included into the Parallel-META pipeline.

## Background

The total number of microbial cells on earth is huge: approximate estimation of them is 10^30 ^[1], and the genomes of these vastly unknown communities of microbes might contain a large number of novel genes with useful functions. However, more than 99% of microbe species were unknown and un-cultivable [2], making traditional isolation and cultivation process non-applicable. Analysis of their metagenomic data is the direct and efficient way to analyse all microbes in the community [3]. The metagenomic approach has made it possible better understanding of microbial diversity as well as their functions. And the broad applications of metagenomic research, including environmental sciences, bioenergy research and human health, have made it an increasingly popular research area.

Metagenomics researches were based on sequencing data from 16S rRNA amplicon, or large-scale shot-gun whole-genome metagenomic sequencing. Early 16S rRNA-based metagenomic survey of microbial communities focused on 16S ribosomal RNA sequences which are relatively short, often conserved within a species, and different between species. The16S rRNA-based metagenomic survey has already produced data for analysis of microbial communities of Sargasso Sea [4], acid mine drainage biofilm [5] and human gut microbiome [6]. Facilitated with Next-Generation-Sequencing (NGS) techniques [7], current metagenomic research has been advanced rapidly. NGS techniques could produce millions of reads at very high speed with relatively low price, thus it enables sequencing at much greater depth. Based on NGS techniques and high performance computational analysis methods, many large-scale metagenomic research projects have been conducted [8], thus made the large-scale metagenomic research the mainstream. In this paper, we were focusing on data analysis for shot-gun whole-genome metagenomic sequencing, in which computational methods play very important roles, especially the similarity-based database search.

The primary goal of metagenomic research is the assessment of taxonomic and functional diversity of microbial communities. Based on NGS data, metagenomic data analysis is both data- and computing-intensive. Therefore, high-performance computing is needed for metagenomic data analysis, especially for projects involving many metagenomic samples.

Traditional high performance computing platform only use CPU cluster. For high computing speed, CPU computing platform always has large amount of high performance CPUs, which also accompanied with high cost and high power consumption. However, with the increase of data size, it becomes more and more difficult for current CPU cluster to satisfy the requirement of the fast-developing metagenomic research. The computing speed of metagenomic data analysis would be accelerated significantly by the combination of GPU computing and parallel CPU computing. For GPU computing, the GPGPU(General Purpose Graphic Process Unit) hardware and CUDA(Compute Unified Device Architecture) software would be the method of choice. CUDA is a massive parallel computing architecture model. Based on nVIDIA (Santa Clara, CA) GPGPUs and SIMT(Single Instruction Multiple Threads), it enables dramatic increases in computing performance by parallel computing with huge number of stream processors. For parallel CPU computing, multi-core CPU could be utilized by implementation of multi-threaded parallel programming.

**Figure 1:**
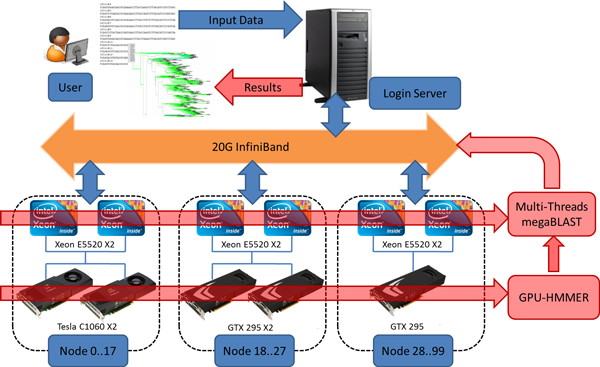
**Parallel computing platform based on GPU and multi-core CPU hardware as well as software pipeline**.

In this work, we used both GPU and multi-core CPU to implement the parallel computing to accelerate the computation. We have proposed a high-performance computational pipeline (Parallel-META) for metagenomic research that has the major advantage of efficient process of large metagenomic dataset. The whole system is illustrated in Figure 1. There were two major components of the system: Multi-core CPU and GPU computing facilities, which enabled the hardware support for parallel process of large metagenomic datasets; and high-performance metagenomic data analysis pipeline, which enabled the software support for parallel metagenomic data analysis. Additionally, the Parallel-META pipeline support advanced metagenomic data analysis functionalities such as the comparison and visualization of multiple samples.

## Methods

### Metagenomics

Large databases of reference sequences, such as Greengenes [9], SILVA [10] and RDP [11] already exist for metagenomic sequence analysis. As most of the microbial communities are still unknown, these databases are also updating frequently. For computational analysis of metagenomic data, the most important tasks include taxonomic and functional analyses. A crucial step in the taxonomic analysis of large-scale metagenomic data is "binning", in which the metagenomic sequences were assigned to phylogenetic groups according to their taxonomic origins at different resolutions: from "kingdom" to "genus" level. There are two categories of binning methods: similarity-based methods that align reads to reference databases, and composition-based methods that use composition patterns (GC content, k-mer frequency, etc.) to cluster reads. The similarity-based methods classify sequences based on sequence homology, which is determined by reference database searches using general purpose alignment tools such as BLAST [12]. The most frequently used similarity-based metagenomic data binning methods include MEGAN [13], CARMA [14] and Sort-ITEM [15]. Most of these software could be used on PC workstation. However, similarity-based methods rely on reference databases that contain sequences of known genomes, so these methods cannot classify the majority of sequences that were from unknown genomes without close references. In contrast, composition-based methods analyse intrinsic sequence features such as GC content, codon usage and k-mer frequency, and compare these features with reference genome sequence of known taxonomic origins. The most frequently used composition-based metagenomic data binning methods include TETRA [16] and PhyloPhythia [17]. Recently, some all-in-one metagenomic data analysis pipelines were introduced, such as Phyloshop [18] and QIIME [19]. The web-based metagenomic annotation platforms, such as MG-RAST [20] and CAMERA [21] were also designed to analyse metagenomic data. However, the increasing number of metagenome data analysis projects needs more and more computational power, which become an increasingly large huddle for the efficient process of metagenome datasets by current pipelines.

### GPU computing

CUDA is a massive parallel computing model based on GPGPU (GPU for short) to solve the rapid increasing data computing problem. It is presented by nVIDIA in 2006 with the G80 series GPU. Different form the traditional GPUs, which are consisted of rendering pipeline of Vertex Engine and Pixel Engine, a CUDA enabled GPU is composed of several SMs (Stream Multiprocessors). The amount of SM depends on the model of GPU. For example, nVIDIA Tesla has 30 SMs, and nVIDIA Quadro FX 880M has 6 SMs. In a single SM, there are also several stream processors and a shared memory which can be accessed by these processors in the same SM. For G80/GT100/GT200 series GPU, one SM is composed by 8 stream processors. The latency of the shared memory is quite low, so it is always used as cache. There is also an on-board memory (Global Memory) which can be shared by all the stream processors in a GPGPU. As GPU cannot directly access the RAM of a computer system, data should be transferred from RAM to Global Memory before GPU computation.

**Figure 2:**
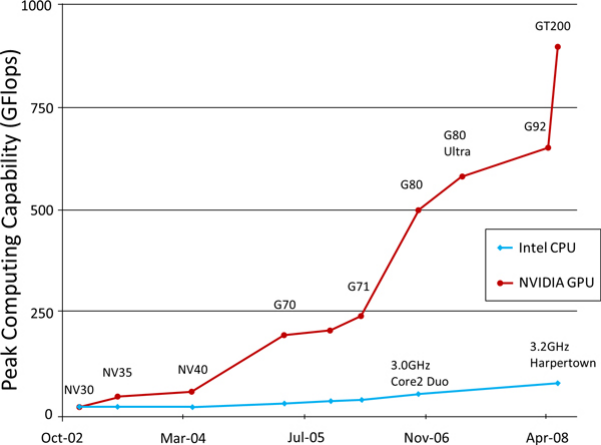
**The peak computing capability comparison between CPU and GPU**.

Based on SIMT (Single Instruction Multiple Threads) structure, GPGPU can invocate a block of threads on one single SM. Each thread performs a single computation on one stream processor. For one block, the maximum number of thread is 512 for the GPU with computation capability 1.X and 1024 for the GPU with computation capability 2.0. Therefore, Total thread number = (Number of Threads in one single Block) × (Number of Blocks). This number can be very large as there might be huge number of SMs in one GPU, meaning that many threads can be executed parallelly at the same time. That is the main reason for the high computing capability and throughput of GPU rather than CPU (Figure 2).

In a computer system equipped with GPU, the CPU system is called host, and the GPU system is called device. CUDA provides a series of APIs which can be invocated by host programs. As GPU cannot directly access the system memory of CPU and Hard disk, data should be transferred from the system memory (RAM) to the Global Memory of GPU by CUDA APIs. Then the stream processors of GPU can exchange data with the Global Memory and Shared Memory.

### Multi-core CPU computing

CPU core is the key part made of Monocrystalline silicon on which instructions can be executed. Before 2005, for normal CPUs, there was only 1 core on one single CPU chipset, which limited the development of the computing capability and efficiency. Engineers used the method that integrating several CPU cores on a chipset to solve these problems. Instructions can be executed on those cores parallelly at the same time. This method not only enhanced the computing capability of CPU, but also reduced the TDP (Thermal Design Power) for cutting down the working voltage and clock rate of CPU cores and the application of power management technology.

The latest architecture of Intel multi-core CPU is Nehalem. Nehalem Xeon 5000 series have such architecture features: (a) four cores integrated into one CPU, (b) hyper-threading technology supports 8 threads at most, (c) each core has a 64 KB L1 cache and 256 KB L2 cache, with an 8 MB L3 cache is shared by all cores and (d) Turbo Boost technology, dynamically adjusts the work frequent of cores.

**Figure 3:**
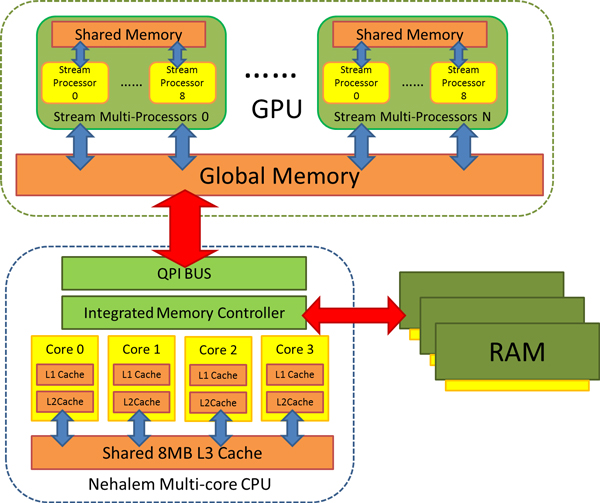
**The architecture of GPU & CPU and data transfer in a computer system**.

A computer system with both GPU and multi-core CPUs is illustrated in Figure 3. This would be a typical hardware architecture for next-generation high performance biological data analysis system, based on which we are testing the Parallel-META metagenomic data analysis pipeline.

### Hardware architecture

In this work, the hardware used was one single node of the GPU computing platform of QIBEBT, CAS (Qingdao Institute of Bioenergy and Bioprocess Technology, Chinese Academy Sciences) computing platform that had the following configuration: CPU: Dual Intel Xeon X5645 2.66 GHz with 12 cores, GPU: nVIDIA Tesla C2070 with 448 processors and 6G DDR5 ECC on board memory, RAM: 72 GB RDIMM DDR3. For this system, the total float computing capability of CPU is 89.6Gflops, and the total float computing capability of GPU is up to 1Tflops.

### Software architecture

Parallel-META is an integrated metagenomic data analysis pipeline developed by QIBEBT, CAS which enables parallel analysis of large metagenomic data and comparison among multiple samples. This pipeline includes four steps: (1) 16S rRNA extraction part: to predict 16S rRNA fragments from metagenomic sequences by HMM (Hidden Markov Model) search of HMMER [22] and then extract out those fragments based on the results of prediction, (2) 16S rRNA mapping part: to map the 16S rRNA fragments extracted by last step onto the database of Greengenes [9] core set using megaBLAST [23] as the alignment tool for their identification, (3) Classification part: to classify the 16S rRNA fragments based on their assignment of the taxonomical terms and mapping to the phylogenetic tree of each sample, (4) Multi-sample comparison part: to compare the taxonomical structure of all samples on different biological levels. After these steps, Parallel-META reports the classification, length distribution, summary of the taxonomic assignments of 16S rRNA fragment sequences and structure difference at different phylogenetic levels among all samples.

**Figure 4:**
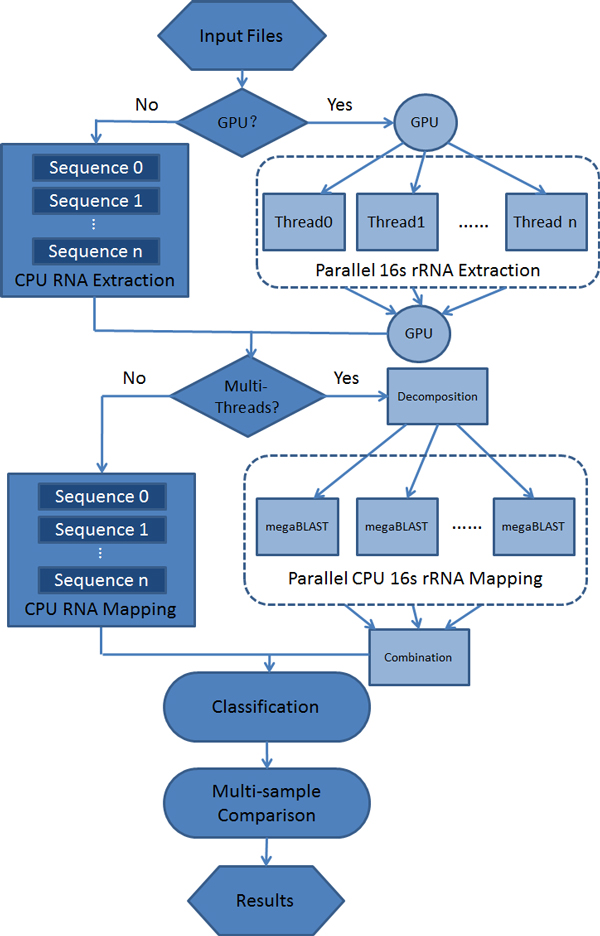
**Metagenomic data analysis pipeline by high-performance parallel computing**.

The key to the efficient and parallel process of large metagenomic data is the parallelization of sequence data binning by database search. To speed up the metagenomic data analysis process, the Parallel-META pipeline is optimized by decomposing large problems into smaller size sub problems and solving them parallelly at the same time on high performance computing devices. Parallel-META mainly optimized the 16S rRNA extraction part and 16S rRNA mapping part. The overall pipeline design was illustrated in Figure 4.

In the 16S rRNA extraction part, we used GPU-HMMER [24] component to implement parallel 16S rRNA prediction on both original sequences and complement sequences instead of traditional HMMER which is based on CPU. The core of HMM search is the Viterbi Algorithm, which is used to compute the most probable path through a given state HMM. Different from CPU computing that performs HMM search by serially executing the loops of Viterbi Algorithm. In GPU-HMMER, the loops are parallelized and expanded into some sub processes. Then each process was mapped to a thread on a stream processor of GPU. As GPU enables the activities of huge number of threads at the same time, Viterbi Algorithm can be done in a much shorter time on GPU than on CPU. Similarly, when computing the complement sequences from the original sequences, each single sequence was also mapped to one stream processor and large number of sequences can be transformed at the same time.

The next step -16S rRNA mapping has been divided into three parts: Problem Decomposition, Parallel Computing, and Result Combination. In the first part, the output data gained from the 16S rRNA extraction were decomposed into sub data files with similar size. Then in the second part, each thread could directly find its input data from the original file and perform the megaBLAST search parallelly by multi-threads programming on CPU. After that, sub results were merged together to get the final result.

After the two steps above, Parallel-META will parse the result of mapping, classify the 16S rRNA fragments of each sample, and then compare the construction of each sample on different biological level. For classification, all samples will be mapped onto one phylogenetic tree with the percentage of each sample on each level. Finally Parallel-META will visualize the common phylogenetic tree of all samples and the comparison of all components with the normalized proportions.

## Results and discussions

We have used four sets of Illumina Solexa GAIIx sequencing-based metagenome data [25] (Table 1, Dataset 1) to evaluate the metagenomic data analysis performance of Parallel-META. Shotgun pair-end libraries of total saliva genomic DNA was prepared (two from healthy population and the other two from caries-active population). Each metagenomic DNA library was then sequenced on one lane of pair-end 100 bp flow cell on Solexa GA-IIx (Illumina, San Diego, CA, USA). After removing the contaminating reads from human hosts, over 7.5 million reads were produced for each of the healthy saliva microbiome, and over 28 million reads were generated for each of the caries-active microbiome. All of the Solexa reads were mapped against the 44 oral reference genomes in Human Microbiome Project [26] to assess the coverage and abundance of these sequenced isolates or their close neighbours in saliva microbiota. These 4 input files were used for checking the performance of analysing different number (from 7.5 million up to 34.4 million) and type of sequences of the optimized pipeline.

**Table 1:** Statistics of metagenomic datasets (Dataset 1)

Type	Sample	Size(MB)	Sequences	16S rRNA number
healthy	Input 1	531.86	7,544,950	2,406
healthy	Input 2	1,576.96	17,591,235	2,118
caries-active	Input 3	2,775.04	34,405,667	6,468
caries-active	Input 4	2,928.64	28,854,628	17,119

In addition, we used 1,968 sets of 16S rRNA targeted sequences [27] (Dataset 2) with 68,667,837 sequences and total size of 11,878.4 MB to test the performance of 16S rRNA targeted sequences analysis. The 16S rRNA sequences were generated from the largest human microbiota of two individuals at four body sites over 396 time-points by Illumina GAIIx.

In the experiments, Firstly, we measured the speed-up of Parallel-META based on GPGPU and multi-core CPU against serial version on single core of single CPU on Dataset 1. Secondly, we tested the Parallel-META pipeline on Dataset 2. Finally, we analysed the comparison result and complete common phylogenetic tree by using Dataset 1. All experiments were performed on one single node of the GPU computing cluster with dual Intel Xeon X5645 CPU (12 cores and 24 threads in total), 72 GB DDR3 RDIMM RAM, and nVIDIA Tesla C2070 GPU(448 stream processors and 6 GB on board memory).

### Results on metagenomic data analysis (Dataset 1)

We ran the Parallel-META with each metagenomic sequence file as input by parallel mode using GPGPU and multi-core CPU and serial mode using one single core of single CPU to compare the speed of two different methods. To reduce the effect of system-wise randomness and noises on the results, each input data were executed three times to get the average results, and the average results were compared. For metagenomic data, we test the performance in 2 steps:

### Step 1 - 16S rRNA extraction

**Figure 5:**
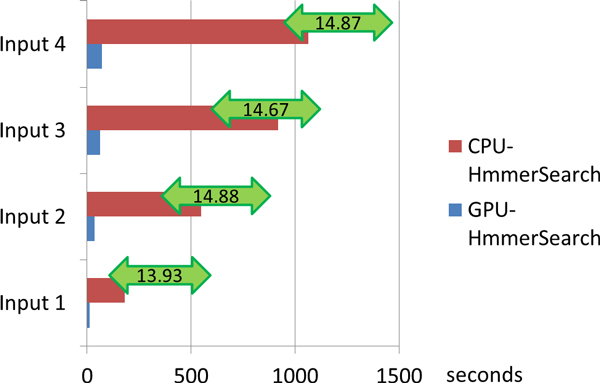
**Comparison of 16S extraction running time of CPU and GPGPU**.

From the results (Figure 5), it was clear that a speed-up of at least 13 have been achieved on each input file in the 16S rRNA extraction part.

Then we have compared the speed-up of input file with increasing file sizes. From Figure 5, we could observe that for input file 1 the speed-up was a little smaller than other input files. This might be due to the fact that for the input file 1 the data size was small. In this situation, the data transfer between Global Memory and RAM became a more significant bottleneck than computing. With the increase of the input file size, the computation proportion also became larger, and the data transfer process has less effect on the whole process. The maximum speed-up rate was 14.88. To get the weighted average speed-up, we used the formula as below:

(1) $$\text{Weighted Average}=\sum_{j=1}^{M}Ni*Si/\sum_{i=1}^{M}Ni$$

Here *N_i _*and *S_i _*were the sequence number and speed-up of input i, and M represented the sample number which is 4 in this experiment, respectively. By this we can get the average speed-up of GPGPU based 16S rRNA extraction of 14.71.

### Step 2 - 16S rRNA mapping

**Figure 6:**
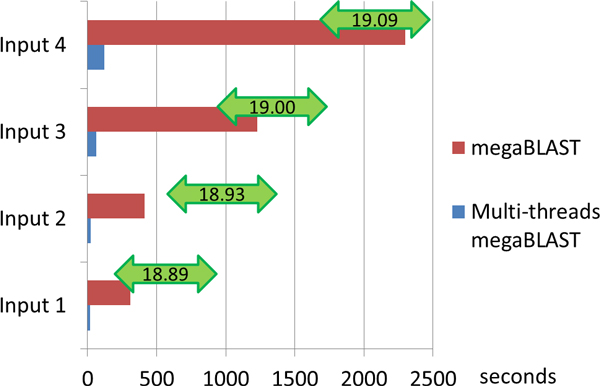
**Comparison of 16S rRNA mapping by Normal and Multi-threads megaBLAST on input files**.

In the experiment on 16S rRNA mapping step, for each input we decomposed the data into sub input files. The number of the sub input files has been designed to be the number of CPU threads. The CPU of the computing platform is Dual Intel Xeon X5645 with 12 cores and 24 threads in total; therefore each input data file was divided into 24sub files, and then each sub problem could be solved on one single thread. From the results (Figure 6), it was clear that a speed-up of 18 and above have been achieved on each input file. We also observed that with the increase size of the input data, the speed-up rate also increased, though such increase was not significant.

In theory, to decompose the input data into 24 parts and parallelly solving them will reduce the runtime to 1/24. However, for the implementation of the multi-thread computing, the time cost of problem decomposition and results combination should also be taken into account. In addition, as the CPU system only has 12 physical CPU cores, if the computing throughput was larger than the CPU computing capability, CPU would use the transition algorithm to automatically manage these threads and some threads maybe executed serially when the CPU was very busy. The maximum speed-up was 19.09, and to get the weighted average speed-up, we used a formula which was similar to the one in 16S rRNA extraction part:

(2) $$\text{Weighted Average}=\sum_{j=1}^{M}Ri*Si/\sum_{i=1}^{M}Ri$$

Here *R_i _*and *S_i _*were the 16S rRNA number and speed-up of input i, and M represented the sample number which is 4 in this experiment, respectively. Therefore, the average speed-up of the16S rRNA mapping was 19.00.

### Overall performance

**Figure 7:**
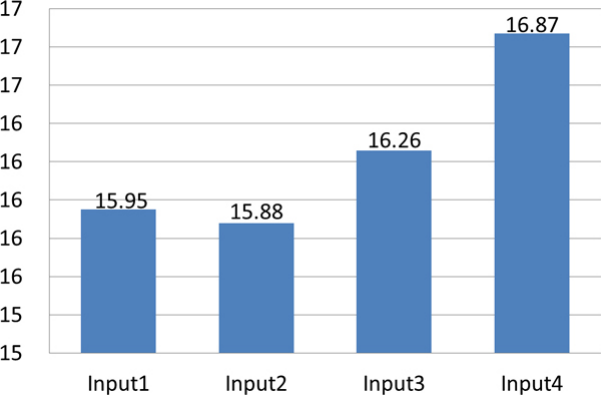
**Total speed-up of Parallel-META compared to single CPU for test datasets in table 1 (dataset 1)**.

Combining these optimization steps, a total speed-up of up to 16.87 has been observed compared to traditional CPU-based methods (Figure 7). More importantly, on all of these datasets, the final results of Parallel-META were identical to the results of the original single CPU-based pipeline, and the taxonomical analysis results were also consistent with the metagenomic data analysis results solely based on 16S rRNA [25].

### Results on massive 16S rRNA data analysis (Dataset 2)

We made the 1,968 16S rRNA sequence files as input files for Parallel-META to evaluate the performance of 16S rRNA targeted sequences analysis. Rather than predicting and extracting 16S rRNA from metagenomic data first, for targeted sequences, Parallel-Meta will skip the 16S rRNA extraction step in the whole pipeline and then continue the last steps.

As the input sequence count quite huge (more than 60 million), this experiment can indicate the computing capability of Parallel-META on massive input data. We also executed each input data three times to get the average results to reduce the effect of system-wise randomness and noises on the results. For the 1,968 input files with 68,667,837 16S rRNA sequences, the average total analysis time of Parallel-META was 6,073 minutes and 51.918 seconds.

### Multi-sample comparison

Given multiple samples, Parallel-META could parse the result of all samples together and map the classification information of one common phylogenetic tree. In the consensus phylogenetic tree, every level of the nodes represents one biological level, therefore from root level to leaf level there are at most six levels which represent phylum, class, order, family, genus and species. For visualization, after the name of each node of the phylogenetic tree there is a bar-chart indicating percentage distrubution of every input samples on this node.

**Figure 8:**
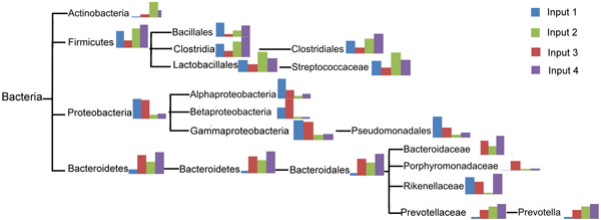
**Common phylogenetic tree with component rate information on different biological level**.

Figure 8 is the consensus phylogenetic tree of four test data sets in table 1 with major components. In the bar-chart of each node, four different colors represent four different datasets of table 1. These results showed the different proportion of 16S reads for each specific taxonomical term from different samples, and indicated the difference in community structures for different metagenomic samples.

## Conclusions

Traditional metagenomic data analyses were conducted on single PC or CPU cluster, based on which handling multiple large metagenomic datasets is becoming more and more difficult. In this work, we have tried to utilize GPU computing and multi-core CPU computing to boost the speed of metagenomic data analysis, and proposed a novel pipeline that enabled the parallel processing of large metagenomic datasets. The Parallel-META pipeline has been applied on several metagenomic data analysis projects for human-associated bacterial communities, such as oral disease-causing microbial community analysis [28]. Several folds of speed-up have been observed, while the sensitivity and discrepancy power were not compromised. With current 10 to more than 15 times of speed-up, some deeper analysis of the metagenomic data, such as the comparison of different samples, would be feasible.

Current Parallel-META pipeline could be improved in different ways. Firstly, the megaBLAST search part could also be implemented on GPU architecture, so that the efficiency of this time-consuming part could be further improved. Secondly, as metagenomic datasets are of different types and sources, the parameters for analysis would be different for each metagenomic dataset. These parameters could be trained based on running Parallel-META on a large amount of different metagenomic datasets, which in turn could improve the accuracy of Parallel-META. Thirdly, the Parallel-META framework could be extended to work with multiple search engines and databases so as to be applicable to different types of metagenomic datasets. Finally, as a general-purpose metagenomic data analysis pipeline, Parallel-META could also incorporate component-based binning methods, which might also significantly improve the speed for clustering metagenomic short reads [29].

Compliment to the high-performance computational pipeline is the high-performance database management system. The high-performance database management system would not only store large amount of results by high-performance computational pipeline, but also facilitate deeper data mining of metagenomic data. Such high-performance database management system would also be incorporated into the next-generation high-performance computational platform for metagenomic data analysis.

## Competing interests

The authors declare that they have no competing interests.

## Authors' contributions

Conceived and designed the experiments: KN and XS. Performed the experiments: XS. Analyzed the data: XS. Contributed reagents/materials/analysis tools: JX, KN and XS. Wrote the paper: KN and XS.
